# COVID-19 incidence and mortality in the Metropolitan Region, Chile: Time, space, and structural factors

**DOI:** 10.1371/journal.pone.0250707

**Published:** 2021-05-06

**Authors:** Pablo Villalobos Dintrans, Claudio Castillo, Felipe de la Fuente, Matilde Maddaleno

**Affiliations:** 1 Programa Centro Salud Pública, Facultad de Ciencias Médicas, Universidad de Santiago, Estación Central, Santiago, Chile; 2 Departamento de Enfermería, Facultad de Medicina, Universidad de Chile, Independencia, Santiago, Chile; University of Essex, UNITED KINGDOM

## Abstract

Demographic, health, and socioeconomic factors significantly inform COVID-19 outcomes. This article analyzes the association of these factors and outcomes in Chile during the first five months of the pandemic. Using the municipalities Metropolitan Region’s municipalities as the unit of analysis, the study looks at the role of time dynamics, space, and place in cases and deaths over a 100-day period between March and July 2020. As a result, common and idiosyncratic elements explain the prevalence and dynamics of infections and mortality. Social determinants of health, particularly multidimensional poverty index and use of public transportation play an important role in explaining differences in outcomes. The article contributes to the understanding of the determinants of COVID-19 highlighting the need to consider time-space dynamics and social determinants as key in the analysis. Structural factors are important to identify at-risk populations and to select policy strategies to prevent and mitigate the effects of COVID-19. The results are especially relevant for similar research in unequal settings.

## Introduction

The novel coronavirus, known as Severe Acute Respiratory Syndrome 2 (SARS-Cov2), firstly described in China at the end of 2019, has produced the new coronavirus disease (COVID-19), declared as a pandemic by the World Health Organization (WHO) on January 30, 2020 [1, 2]. By July 31, 2020, this pandemic has caused over seventeen million confirmed cases and 668,910 deaths worldwide [3].

**Fig 1:**
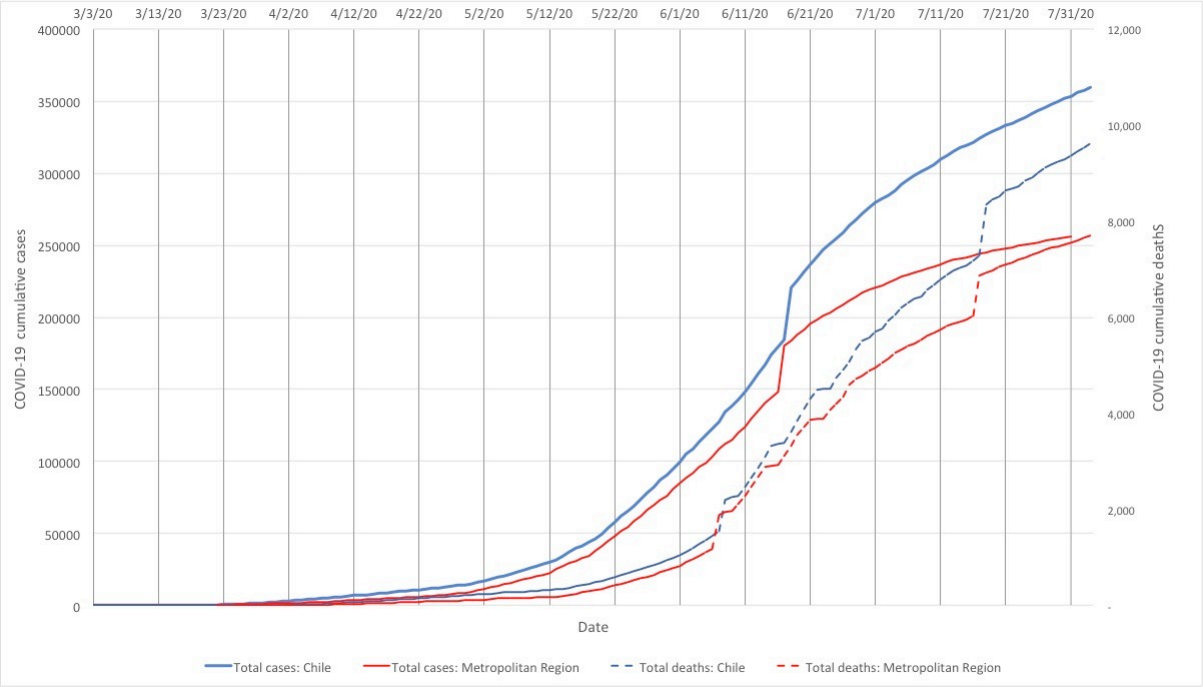
Evolution of cumulative cases, deaths, and policy milestones in Chile and the Metropolitan Region (March-July, 2020).

The Americas currently face the heaviest burden of the pandemic and Chile has been one of the countries more affected by this new virus [3]. Despite implementing testing, contact tracing, isolation practices, health messaging, and lockdown efforts [4, 5], Chile reached 395,261 cases by July 30, 2020 and had one of the highest mortality rates, globally [6, 7]. Fig 1 presents the evolution of policy and epidemiological events in the country and the Metropolitan Region over the period of study. Both cases and deaths at national level resemble the pattern in the Metropolitan Region, with cases and deaths increasing even after measures were applied.

Several studies on the determinants of COVID-19 have highlighted the multicausal nature of the disease. Research has investigated the impact of countries’ demographic profile, the effect of preexisting health conditions, and the impact of socioeconomic factors in explaining COVID-19-related outcomes [8–13]. Chilean COVID-19 reports, thus far, have incorporated demographics and health conditions [13]. However, the links with social determinants of health have been less studied in the country. Moreover, other relevant features of the disease, such as its temporal and spatial dynamics, are almost completely absent.

This study analyzes demographic, health-related, and socioeconomic factors on COVID-19 infections and deaths in Chile. We use the 52 administrative units (municipalities) in the Metropolitan Region of Chile. The Metropolitan Region provides unique research and health-care policy opportunities as it: (a) is highly-populated, accounting for 40.5% of the Chilean population including that of the capital; (b) accounts for 71.91% of cases and 74.58% of deaths as of July 31, 2020, nationally; and (c) exhibits larger cumulative incidence rates (3491.3 vs 2031.3) and mortality rates per 100,000 people (63.7 vs. 30.7) [13, 14]. Finally, the Metropolitan Region’s particular spatial characteristics create a case for analysis. The 52 municipalities are spread over a relatively small area and most municipalities are highly connected, creating an area where administrative boundaries exist but within a large conurbation area.

On May 15th, 2020 quarantine started in 38 out of the 52 municipalities of the Metropolitan Region. The Chilean government established the concept of dynamic quarantine, which implies that some municipalities may or may not be in quarantine depending on each administrative district’s epidemiological characteristics. Curfew is still applied in all the territory. This situation stays as by February 2021.

Additionally, COVID-19 outcomes and variables of interest, particularly sociodemographic variables, are not randomly distributed within the Metropolitan Region. People cluster based on socioeconomic and demographic characteristics, such as income and age [15–19]. Marked patterns of residential segregation and geographical concentration of income exist in Chile, particularly in the Metropolitan Region [19–23]. Evidence also exists of regional inequities in access to healthcare [24–30]. Space and place have increasingly been used to analyze and understand health decisions and outcomes [31, 32]; this relationship has already proven to be important for the new COVID-19 disease [33–38]. Understanding the context of an unequal and segregated city as it relates to infection and death patterns can inform public, health-worker, and policymaker support for prevention and community-tailored interventions. The information can also serve other countries that, like the Metropolitan Region in Chile, are navigating COVID-19 consequences and inequities.

## Materials and methods

### Data sources and variables

#### Dependent variables

The study proposes the analysis of the interactions of determinants and COVID-19 outcomes at the municipality level. To carry out this analysis, the study uses two groups of dependent variables: COVID-19 cases and deaths. Both variables are relevant: cases relate to the ability to prevent infection in each municipality, deaths reflect individual response to the virus. Both are associated with the environmental context, including the health system’s capacity to manage.

Each variable was measured considering two perspectives: level and change. Level variables capture how the “length” of COVID-19 impacts the population in a geographical area, while the change variables capture the “speed” at which COVID-19 affects an area. Assessing speed adds depth of understanding as quick outbreaks signal municipalities’ vulnerability, inability to prevent the spread of the virus, and can be relevant to understand patterns in deaths, e.g. if sudden outbreaks overburden or collapse the healthcare system. Table 1 summarizes this information.

**Table 1:** Indicators, dimensions, and dependent variables.

Indicator/ dimension	Level	Change
Infections	• Cumulative incidence rate (cases) after 100 days	Days to peak (cases)
	• Peak of cases (daily)	
Deaths	• Cumulative incidence rate (deaths) after 100 days	Days to peak (deaths)
	• Peak of deaths (daily)	

Source: Authors.

Six dependent variables of interest were used in the analysis. Cumulative incidence was calculated as the number of COVID-19 confirmed cases per 100,000 people in each municipality. The peak variables were defined as the highest number of current (active) cases according to symptom onset date in each administrative unit. The speed of the impact was measured by the number of days between the first case and the maximum number of cases and deaths registered over the 100-day period for each municipality (see S1 Table). Other measures of speed, such as the average growth rate in the number of cases and deaths, required a level of detail in the data (daily basis information at municipality level) that was not available.

Data on the number of positive COVID-19 cases was retrieved from open-source statistics published by the Chilean Ministry of Science, Technology, Knowledge and Innovation [39]. The number of deaths was obtained from the Information and Statistic Department published by the Chilean Ministry of Health [40].

#### Independent variables

We use three groups of variables to identify the effect of municipality-level features on COVID-19 outcomes: demographic, health, and socioeconomic variables, primarily from the 2017 National Socioeconomic Characterization survey (CASEN). Since 1990, the Chilean Ministry of Social Development periodically conducts the CASEN to assess socio-economic conditions of Chilean families and effects of the country’s social policies. The self-reported survey samples almost 70,000 households from 16 administrative regions. The survey contains 42,601 individual responses from the Metropolitan Region [41].

Demographic indicators included the percentages of women and older people (65+ and 80+) in each municipality, as these variables were associated with COVID-19 outcomes in other countries [42–46]. In Chile, age is highly correlated to COVID-19-related hospitalizations and deaths [13]. Although evidence currently is unclear, the percentage of children could help explain viral spread as studies have found children carry higher viral loads [47, 48]. They also could face COVID-19-related health complications [49, 50]. Additionally, other variables, such as proportion of migrants, the population density, and rurality were included, as they help explain both infection rates and mortality [51–56].

Health-related indicators, including the health system’s features and health outcomes, have been used to identify contagion and mortality patters, primarily through pre-existing health conditions. We, first, included the population covered by the public health insurance, a self-reported variable that captures barriers to healthcare, and a dummy variable that identifies people that live far from a health center (2.5 kilometers or more). We expected these variables to explain mainly COVID-19-related health outcomes (deaths), as they are proxies for healthcare access and quality, and also reflect other broader inequalities across the population. We, then, constructed a dummy variable to capture whether individuals report having at least one COVID-19-related health condition, as defined by the Centers for Disease Control and Prevention (CDC) and the Chilean Ministry of Health, [13, 57, 58].

Finally, several variables that capture the socioeconomic level were considered. Water availability can be seen both as a socioeconomic and health indicator. Given the nature of the virus, access to water is expected to impact people’s preventive behaviors, particularly as hand-washing is a key COVID-19 prevention strategy [59, 60]. Poverty as an indicator captures several risk factors associated both with higher rates of infection and death. Poverty is associated with people’s vulnerability to COVID-19 and impacts the variables of interest through several channels: poor sanitary conditions, access to information, inability to follow prevention strategies—such as hand-washing, the use of facemasks, and social distancing—, and lower access to healthcare, among others [37, 61–64]. The CASEN survey includes a variable called multidimensional poverty, an index of socioeconomic vulnerability that summarizes several aspects related to social determinants and COVID-19 (see S2 Table) [65]. Using this index has advantages and disadvantages. While it simplifies the estimation by capturing several socioeconomic factors into one variable, as used in other studies, it hinders the understanding of the underlying channels through which social determinants impact health outcomes [66, 67]. Consequently, we estimate models including this index and others in which the different dimensions—income poverty, overcrowding, education, health insurance coverage, and job status—are considered independently. All these variables can explain infections and deaths by capturing a household’s structural inability to follow the preventive measures (hand-washing, use of face masks, physical distancing, and quarantine) and seek healthcare (e.g. health literacy, financial feasibility, etc.) [68–73]. Finally, the use of public transportation and the availability of green spaces indicate people’s ability to comply with social distancing strategies [56]. The indicators potentially impact infection rates and could also influence COVID-19 deaths, since they show mobility challenges within the city, a factor that could be relevant in health emergencies [74]. Maps and spatial information were obtained from the website of the Inter-Ministerial Committee on Geographic Information [75].

All dummy variables were transformed to capture the percentage of people reporting each condition in each municipality. Continuous variables represent the municipalities’ average value. The survey’s expansion factor at municipal-level was used to expand the sample’s values to a population estimate.

A summary of the variables used and sources of information is presented in Table 2. For the independent variables, data includes information collected from March 3rd to July 30th, 2020.

**Table 2:** List of variables, definitions and source.

Variable	Variable description	Source
Cumulative incidence rate (cases) after 100 days	Number of confirmed cases of COVID-19 per 100,000 persons per day 100 from the date of notification in the national registry of the first case in each administrative unit (municipality)	Ministry of Science, Technology, Knowledge and Innovation
		Ministry of Health
Cumulative incidence rate (cases) peak of cases (daily)	Number of confirmed cases of COVID-19 per 100,000 people on the day with the highest number of current (active) cases according to onset of symptoms in each administrative unit (municipality)	Ministry of Science, Technology, Knowledge and Innovation
Days to peak (cases)	Number of days between the day of the first case notified in the national registry and the day with the highest number of current cases (active) according to the onset of symptoms in each administrative unit (municipality)	Ministry of Science, Technology, Knowledge and Innovation
		Ministry of Health
Cumulative incidence rate (deaths) after 100 days	Number of deaths due to COVID-19 (with and without laboratory confirmation -U07.1 and U07.2, respectively-) per 100,000 persons per day 100 from the date of notification in the national registry of the first case in each administrative unit (municipality)	Department of Statistics and Health Information (DEIS) Ministry of Health
Cumulative incidence rate (deaths) peak of deaths (daily)	Number of deaths due to COVID-19 (with and without laboratory confirmation—U07.1 and U07.2, respectively) per 100,000 people during the day with the highest number of deaths in each administrative unit (municipality)	Department of Statistics and Health Information (DEIS) Ministry of Health
Days to peak (deaths)	Number of days between the day of the first case notified in the national registry and day with the highest number of deaths due to COVID-19 (with and without laboratory confirmation—U07.1 and U07.2, respectively) in each administrative unit (municipality)	Department of Statistics and Health Information (DEIS) Ministry of Health
Women	Percentage of women living in the administrative unit (municipality)	Casen 2017
Children	Percentage of children under age of 6 living in the administrative unit (municipality)	Casen 2017
People 65+	Percentage of people 65+ living in the administrative unit (municipality)	Casen 2017
People 80+	Percentage of people 80+ living in the administrative unit (municipality)	Casen 2017
Migrants	Percentage of people living in the administrative unit (municipality) who declare having a foreign nationality and have been living in another country five years ago	Casen 2017
Population density	Ratio of population of a administrative unit (municipality) and its area in km^2^	National Municipal Information System (SINIM), 2019
Rurality	Percentage of people living in a territory defined as rural in each administrative unit.	National Municipal Information System (SINIM), 2019
Multidimensional poverty	Percentage of people classified as poor using the index of multidimensional poverty, in each administrative unit. Index that measures deficiencies per household in education (schooling (7.5%), attendance (7.5%) and backwardness (7.5%)), health (insurance affiliation (7.5%), malnutrition (7.5%) and access to care (7.5%)), work and social security (occupation (7.5%), social security (7, 5%) and pensions (7.5%)), housing and environment (basic services (7.5%), housing status and overcrowding (7.5%) and environment (7.5%)) and networks and social cohesion (perceived social support and participation (3.33%), equal treatment (3.33%) and security (3.33%)) in the administrative unit (municipality)	Casen 2017
Income poverty	Percentage of people who cannot meet their basic needs, estimated from a basic food basket in the administrative unit (municipality), using the CASEN definition.	Casen 2017
No overcrowding	Percentage of people who declares living in a household with less than 2.5 persons per exclusive use bedroom in the administrative unit (municipality)	Casen 2017
Critical overcrowding	Percentage of people who declares living in a household with 5 and more persons per exclusive use bedroom; and households without exclusive use bedrooms in the administrative unit (municipality)	Casen 2017
Years of education	Average number of years of schooling of the population aged 15 and over in the administrative unit (municipality)	Casen 2017
Self-employed worker	Percentage of people who declares their job status being self-employed worker in the administrative unit (municipality)	Casen 2017
Green spaces	Ratio of green area (m^2^) per person living in the administrative unit (municipality)	System of Urban Development Indicators and Standards (SIEDU) of the National Institute of Statistics
Use public transportation	Percentage of people in each administrative unit (municipality) that declares using public transportation regularly use public transportation	Casen 2017
Water inside the house	Percentage of people that declares having public water with an in-home tap in the administrative unit (municipality)	Casen 2017
Public health insurance	Percentage of the population that is covered by health insurance (public, private or armed forces) in the administrative unit (municipality)	Casen 2017
Difficulty getting healthcare	Percentage of the people that claims to have had a problem obtaining care (any of the following reasons: had no time, had no money, it is too expensive, asked but did not get an appointment with the physician, or got an appointment but further in time), in the last three months in the administrative unit (municipality)	Casen 2017
Distance to health center	Percentage of the people in each administrative unit (municipality) that declares living at less than 2.5 kilometers from a health center	Casen 2017
COVID-19 risk factors	Percentage of the population that declares to have been in medical treatment during the last twelve months for arterial hypertension, diabetes, acute myocardial infarction, chronic obstructive pulmonary disease, moderate or severe bronchial asthma or terminal chronic renal failure in the administrative unit (municipality)	Index created from Casen 2017

The selected variables cover an ample spectrum of dimensions (demographic, health, and socioeconomic factors) related to the impact (infection and deaths) of COVID-19. In terms of the spatial analysis, they include variables to explain geographical variations based on compositional issues (i.e. differences in the kind of people who live in each place) as well as contextual explanations (i.e. differences between the places), understanding that both are relevant in the relationship between health and place [76].

#### Data analysis

To acknowledge the temporal dynamics of the disease, the study uses a standardized 100-days period since the first case was reported in each municipality. As for the role of space and place in explaining COVID-19 cases and deaths, we used a spatial analysis approach to look at the data. To identify the determinants of infection and mortality due to COVID-19 in the Metropolitan Region in Chile, we carried out multivariable regressions to explain the set of dependent variables, using the three groups of explanatory variables described above. As previously stated, one of the most salient features when performing municipality-level analysis in the Metropolitan Region in Chile is the presence of geographic clusters, particularly when looking at socioeconomic indicators. Considering the way in which COVID-19 is transmitted and the potential differences in access and quality of healthcare, we expect space and place to play an important role in determining both values in the independent and dependent variables, as well as their interactions, justifying the use of spatial analysis.

The first step in the spatial analysis is the definition of neighbors. In this case, we used a first-order queen contiguity matrix, i.e. we defined neighbors as all municipalities that share a border. Given the nature of the data—the existence of clusters in different areas of the region and the high heterogeneity in the size of the municipalities—a distance-based approach was discarded [77]. Analysis was also carried out using the k-nearest neighbors criterion (using the average number of contiguous neighbors, 5 neighbors) and main results hold.

To understand the impact of different variables in the incidence and mortality due to COVID-19 in the region, we use ordinary least squares (OLS) regressions. We estimate several models using an exploratory approach to the data. If space and place are relevant in determining the variables in our analysis, then the errors in the OLS regressions are spatially-correlated and, consequently, the results are biased [78, 79]. To test the presence of spatial correlation on the OLS regression, the global Moran’s I test—that indicates both the existence and degree of spatial autocorrelation [79]—is applied to the regression’s residuals, although there are several ways to perform this test [79–81]. The statistics is used to test the null hypothesis of spatial randomness, showing whether the residuals are randomly distributed [82, 83]. If the null hypothesis is rejected, the OLS analysis needs to be adjusted to consider the spatial effects. There are two main strategies to estimate spatial autoregressive models: spatial lag and spatial error. The spatial lag model (also known as contagion model) incorporates space as a right hand-side variable, estimating a coefficient for the spatial effect; the error model does not incorporate spatial as a covariate, but includes it in the structure of the residuals [84–86]. Conceptually, spatial lag models seem more appropriate to adjust for spatial autocorrelation in the case of infections, since it is expected that the number of COVID-19 cases in one municipality affect the cases in the neighboring areas, the contagion effects. However, this is not necessarily true for mortality, particularly once controlling for the case incidence rate. In this case, a spatial error model appears more suitable, since unobserved spatial effects are expected to drive the spatial autocorrelation in the residuals. Consequently, spatially correlated regressions of cases are adjusted using a spatial lag model, and an error model is utilized for the death regressions.

Incidence and mortality data were collected and calculated using Microsoft Excel. Descriptive statistics and multivariable regressions were estimated using STATA, and spatial analysis and visualizations were conducted in GeoDa.

## Results

### Data and spatial tests

Table 3 shows the descriptive statistics of the sample. First, it is observed the large heterogeneity in most variables between municipalities, both in the dependent and independent variables. As stated before, this reflects the different realities within the Metropolitan Region, as well as the differences in terms of COVID-19 outcomes. Second, spatial autocorrelation appears as statistically significant and positive for all the variables of interest (different measures of infection and mortality), as well as many of the independent variables, particularly those related to social determinants of health, showing that several of the variables of interest tend to cluster spatially.

**Table 3:** Descriptive statistics.

Variable	Average	St. Dev.	Min	Max	Moran’s I
Cumulative incidence rate (cases)	2,477.00	901.02	1025.10	4,788.00	0.43***
Peak of cases	2,106.99	857.47	588.00	3,983.90	0.43***
Days to peak of cases	93.00	11.53	65.00	118.00	0.16**
Cumulative incidence rate (deaths)	92.55	45.39	25.10	224.25	0.46***
Peak of deaths	50.79	29.74	3.51	137.85	0.18**
Days to peak of deaths	84.27	15.47	38.00	107.00	0.10*
Women	0.52	0.02	0.46	0.56	0.04
Children	0.07	0.01	0.04	0.09	-0.04
People 65+	0.13	0.04	0.06	0.23	0.26***
People 80+	0.03	0.01	0.00	0.07	0.07
Migrants	0.03	0.06	0.00	0.38	0.26***
Population density	6,165.66	6,152.61	3.73	22,870.32	0.65***
Rurality	0.12	0.21	0.00	1.00	0.48***
Public health insurance	0.75	0.18	0.13	0.99	0.49***
Problems accessing healthcare	0.00	0.00	0.00	0.01	0.03
Distance to health center	0.88	0.13	0.45	0.99	0.29***
COVID-19 risk factors	0.16	0.03	0.07	0.24	0.24***
Water inside the house	0.98	0.02	0.92	1.00	0.02
Multidimensional poverty	0.22	0.08	0.03	0.38	0.30***
Income poverty	0.06	0.03	0.00	0.14	0.26***
No overcrowding	0.89	0.05	0.78	0.98	0.27***
Critical overcrowding	0.01	0.01	0.00	0.05	0.12*
Years of education	5.88	4.14	1.55	18.67	0.47***
Self-employed worker	0.10	0.02	0.05	0.15	0.06
Green spaces	9.63	1.50	8.00	14.05	0.33***
Use of public transportation	0.21	0.07	0.05	0.41	0.49***

All variables are expressed as the share of the municipality’s total population, except for "Population density", "Green spaces", "Years of education" and "Minutes in public transportation" that report the municipality’s average.Min and Max refer to the minimum and maximum values at municipality, not individual level. Significance level

*** p<0.01

** p<0.05

* p<0.1. Moran’s I statistical significance was assessed using pseudo p values with 999,999 permutations.

**Fig 2:**
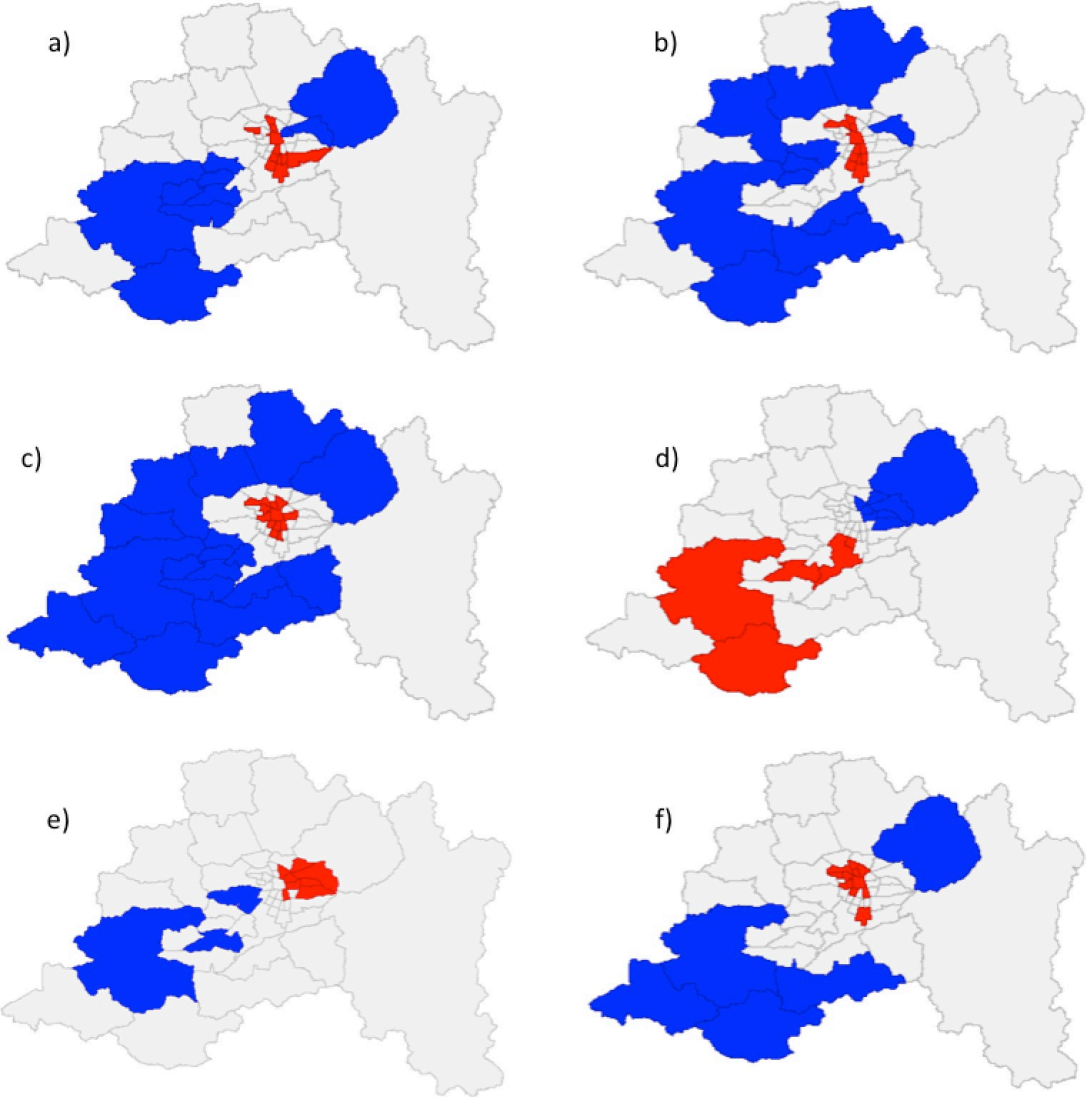
G_i_* tests for clustering in selected variables. (a) infections (100 days cumulative rate); (b) deaths (100 days cumulative rate); (c) population density; (d) public health insurance; (e) average years of education; (f) use of public transportation. Blue: low-value clusters (95% significance); Red: high-value clusters (95% significance); Grey: not significant areas.

This result gives a first warning on the potential effect of space between COVID-19 outcomes and municipal-level features. Spatial autocorrelation and measures can be broadly classified into global and local measures [87]. Moran’s I is a global test that indicates the presence of spatial correlation; a local test can be used to answer where this correlation is. In this case, the Gi*—an statistic that indicates the extent to which a location is surrounded by a cluster of high or low values—is used to identify areas where hot and cold spots detection of selected variables respect to the global average [87, 88]. Fig 2 shows the results for the Gi* tests in selected dependent and independent variables. A common feature in the use of local measures of autocorrelation is multiple and dependent testing: because the same hypothesis is tested several times (and using similar data), statistically significant results will be found just by chance (false discovery rate). In this case, figure shows values without this correction, which can lead to over identification of these clusters [89]. The figure exhibits clusters around the Santiago downtown area vs peripheral municipalities, as well as an east-west pattern, particularly for socioeconomic variables.

#### Infections

To identify how different variables relate to COVID-19 infections and deaths, several multivariable regressions were estimated. Table 4 presents the results for the infection-related dependent variables. As shown in Table 1, each variable has three different ways to be measured: cumulative incidence rate (columns 1 and 2), peak of cases (columns 3 and 4), and days to the peak (columns 5 and 6). Each model is estimated using multidimensional poverty (even columns) and a set of socioeconomic variables (odd columns).

**Table 4:** Infection regressions (OLS).

Variables	Cumulative incidence rate		Peak		Days to peak	
	(1)	(2)	(3)	(4)	(5)	(6)
Women	-5.434	-6.097	4.462	4.158	0.115	0.144
Children	-0.043	-3.667	6.027	0.792	0.0805	0.073
People 65+	6.415	5.517	-2.984	-4.732	-0.154**	-0.135*
People 80+	0.488	6.113	-1.859	1.479	-0.0211	-0.000521
Migrants	-2.751	-5.453	1.504	1.268	0.0231	0.0429
Population density	4.07E-05	6.35e-05*	2.45E-05	4.59E-05	-3.33E-07	-4.92E-07
Rurality	0.89	1.547	-0.176	-0.267	-0.025	-0.044***
Multidimensional poverty	5.065***		4.528***		1.68E-05	
Income poverty		2.433		5.885		-0.0148
No overcrowding		-2.11		2.701		-0.00375
Critical overcrowding		10.98		8.536		-0.0337
Years of education		-0.101		-0.33		0.00116
Self-employed worker		4.467		0.117		-0.198**
Green spaces	0.0232	0.0223	-0.0161	-0.00561	-0.000915*	-0.00069
Use public transportation	9.526***	8.803***	2.537	2.122	-0.0459	-0.0606
Water inside the house	-4.636	-2.112	-0.262	-1.411	0.0485	0.0593
Public health insurance		0.893		-0.86		0.0164
Difficulty getting healthcare	-91.34	-113.2*	30.93	43.17	1.279*	1.957**
Distance to health center	0.441	0.379	1.347	1.194	0.00237	-0.0095
COVID-19 risk factors	0.0994	-3.177	4.436	3.435	0.028	0.0604
Constant	5,196	6,448	-3,440	490.1	12.54	-5.576
R-squared	0.665	0.692	0.609	0.632	0.351	0.428
Adjusted R-squared	0.538	0.509	0.461	0.414	0.105	0.088
Moran’s I (residuals)	-0.0374	-0.0222	-0.0587	-0.0687	-0.0309	-0.0196

Significance level (robust standard errors)

*** p<0.01

** p<0.05

* p<0.1

First, it is noted that the determinants of level and change in infections differ. As expected, the use of public transportation shows a significant and positive association with cumulative incidence rate; results for change-type regression (columns 5 and 6) are less consistent, with the share of people 65+, rurality, self-employment, green spaces, and difficulty to get healthcare showing significant coefficients. Second, multidimensional poverty appears as important to explain the number of cases (columns 1 and 3), while the effect vanishes when looking at the impact of a set of socioeconomic variables instead of the index of socioeconomic vulnerability (columns 2 and 4). The effect, as expected, is also positive showing that multidimensional poverty is a risk factor for COVID-19 contagion at the municipality level. Third, the model’s overall fit is better in the level-type regressions, explaining 60% of the variation in the dependent variables. In the case of estimations 5 and 6, count models using Poisson regression were also estimated; results—in terms of significance and sign of the coefficients—hold when using this alternative. Jarque-Bera tests fail to reject the null hypothesis of normality of errors.

Finally, Moran’s I tests for spatial autocorrelation of the residuals show that the hypothesis that OLS residuals are distributed randomly in the space cannot be rejected for all models.

#### Deaths

Table 5 presents the same set of estimations for the mortality variables. In this case, four different models are estimated for each one of the independent variables, cumulative mortality rate (columns 1 to 4), peak of deaths (columns 5 to 8), and days to the peak of deaths (columns 9 to 12). As before, each model is estimated using either the multidimensional poverty index or a set of socioeconomic factors; additionally models are estimated including and excluding the cumulative incidence rate of cases as explanatory variable (even and odd columns, respectively).

**Table 5:** Deaths regressions (OLS).

Variables	Cumulative incidence rate				Peak				Days to peak			
	(1)	(2)	(3)	(4)	(5)	(6)	(7)	(8)	(9)	(10)	(11)	(12)
Women	-0.231	-0.102	-0.195	-0.030	0.128	0.176	0.002	0.064	-0.074	-0.093	-0.095	-0.096
Children	-0.361	-0.360	-0.347	-0.247	-0.515	-0.514	-0.374	-0.337	-0.129	-0.129	0.004	0.004
People 65+	0.345*	0.193	0.236	0.087	0.056	-0.001	-0.080	-0.136	-0.023	-0.001	-0.091	-0.090
People 80+	0.088	0.077	0.114	-0.052	-0.097	-0.102	-0.036	-0.099	0.304	0.306	0.127	0.127
Migrants	-0.031	0.034	-0.065	0.083	-0.072	-0.047	-0.178	-0.122	-0.090	-0.099	-0.047	-0.048
Population density	3.21e-06**	2.25e-06*	4.60e-06***	2.87e-06*	0.000	0.000	2.50e-06**	0.000	0.000	0.000	0.000	0.000
Rurality	-0.041	-0.0625*	-0.027	-0.069	-0.056	-0.064*	-0.013	-0.029	-0.079**	-0.076**	-0.071*	-0.071*
Multidimensional poverty	0.264***	0.144			0.141*	0.096			-0.029	-0.012		
Income poverty			0.020	-0.047			0.339	0.314			0.033	0.033
No overcrowding			0.158	0.215			0.068	0.089			0.169*	0.169*
Critical overcrowding			0.490	0.192			0.410	0.298			-0.009	-0.008
Years of education			-0.003	0.000			0.000	0.001			-0.001	-0.001
Self-employed worker			0.372	0.250			0.419*	0.374			0.219	0.219
Green spaces	0.001	0.000	0.001	0.000	0.000	-0.001	0.000	0.000	0.000	0.000	0.000	0.000
Use public transportation	0.216	-0.010	0.093	-0.146	-0.008	-0.093	-0.006	-0.096	0.130	0.162	0.129	0.129
Water inside the house	-0.147	-0.037	-0.057	0.001	0.140	0.181	0.110	0.131	0.077	0.061	0.003	0.003
Public health insurance			0.114	0.090			0.002	-0.008			-0.020	-0.020
Difficulty getting healthcare	-1.514	0.651	-2.022	1.050	3.231	4.043*	1.181	2.336	0.918	0.606	0.658	0.655
Distance to health center	0.005	-0.006	0.038	0.028	-0.004	-0.008	0.023	0.020	-0.077**	-0.076**	-0.048	-0.048
COVID-19 risk factors	-0.148	-0.151	-0.218	-0.132	-0.150	-0.151	-0.166	-0.133	-0.081	-0.081	-0.012	-0.012
Cumulative incidence rate (cases)		0.023***		0.027***		0.009		0.010		-0.003		0.000
Constant	234.500	111.300	-49.380	-224.400	-127.800	-174.000	-152.000	-217.800	95.170	112.900	-11.640	-11.480
R-squared	0.747	0.822	0.732	0.821	0.481	0.505	0.523	0.553	0.636	0.642	0.711	0.711
Adjusted R-squared	0.652	0.747	0.572	0.706	0.284	0.299	0.240	0.264	0.499	0.493	0.539	0.525
Moran’s I (residuals)	-0.023	-0.002	-0.054	-0.028	-0.165**	-0.169**	-0.139*	-0.149**	-0.200***	-0.164**	-0.233***	-0.191**

Significance level (robust standard errors)

*** p<0.01

** p<0.05

* p<0.1

The first result is that the determinants of infections and deaths are not the same. However, as expected, some variables seem to explain variation in both types of variables. As in the case of infections, level-type and change-type estimations present common and specific determinants. For deaths, for level-type variables (columns 1 to 8) the share of people over 65 years old, population density, multidimensional poverty, and the prevalence of cases have significant and positive coefficients; overcrowding and distance to a health center also contribute to explain whether a municipality reaches the peak of cases faster or slower. Just like in the case of infection models, multidimensional poverty captures an effect that is not explained by a broad set of socioeconomic factors. The model also does a better job explaining cumulative rates than peaks and days to the peak, and the overall fit (R^2^) is larger than for the infection regressions in Table 4. As before, using Poisson regressions for days to the peak does not change the main results. Jarque-Bera tests fail to reject the null hypothesis of normality of errors. Unlike the infection regressions, in this case, the hypothesis of a random spatial distribution of the residuals is rejected in 8 out of 12 cases, highlighting the need to use spatial regression models to account for the presence of spatial autocorrelation.

### Simplified models and spatial regressions

Based on the results from Tables 4 and 5, a simplified set of regressions is estimated. Some potential problems with inference are related to the degrees of freedom due to a large number of explanatory variables and the relatively small sample (n = 52), as well as the existence of multicollinearity between the variables. As for the previous estimations, these results should be interpreted with a conservative criterion. Table 6 shows these reduced models, based on the previous results (infection and deaths OLS regressions). As emphasized above, both variables (cases and deaths) share some explanatory variables and differ in others. In this case, it is also observed that level-type and change-type models have different determinants. Notably, the overall fit of the model using a reduced set of independent variables is similar to the one obtained in Tables 4 and 5, showing that an important proportion of the variation in the variables of interest can be explained by a small set of variables.

**Table 6:** Simplified regressions (OLS).

Variables	Cumulative infection rate	Peak of cases	Days to peak (cases)	Cumulative death rate	Peak of deaths	Days to peak (deaths)
	(1)	(2)	(3)	(4)	(5)	(6)
People 65+				0.147*	0.0923	0.027
Population density	4.25e-05*	3.19e-05*	-3.82E-07	2.61e-06***	8.96E-07	2.12E-07
Rurality				-0.0568***	-0.070***	-0.114***
Multidimensional poverty	5.691***	5.185***	0.00244	0.115*	0.0905	-0.0212
Use public transportation	4.697***	5.076***	0.0688**			
Difficulty getting healthcare	-31.23	4.56	-0.175			
Distance to health center				-0.00164	-0.0229	-0.0943***
Cumulative incidence				0.0225***	0.00523	-0.000648
Constant	33.57	-280.9	80.76***	-15.59	29.01	155.0***
R-squared	0.568	0.562	0.101	0.805	0.423	0.576
Adjusted R-squared	0.532	0.525	0.025	0.780	0.346	0.519
Moran’s I (residuals)	0.081	-0.087	0.1251**	-0.0056	-0.1118	-0.1418*

Significance level (robust standard errors)

*** p<0.01

** p<0.05

* p<0.1

Finally, considering the presence of spatial autocorrelation in the OLS residuals, spatial regressions are used to control for these effects. As discussed above, a spatial lag regression is used for the infection model (column 1), while spatial error regressions are estimated for death models (columns 2 to 10). In both cases, the same specification used in Tables 3–6 was utilized.

First, adding a spatial dimension removes the spatial correlation in the residuals in six cases where OLS residuals show spatial autocorrelation: days to peak of cases (column 1), peak of deaths (columns 2 to 5), and days to peaks of deaths (columns 6 to 10). However, overall the results improve, reflecting the addition of a previously omitted significant variable. Not only does overall fit increase (R^2^) but results, in terms of individual coefficients, become more consistent. As Table 7 shows, the percentage of children and the rurality reduces the magnitude in the peak of deaths, but reduces the days to the peak. The opposite occurs for multidimensional poverty, again, a risk factor to explain the level and velocity of deaths. The speeding effect is also observed for the percentage of people 65+, migrants, overcrowding, and distance to health centers, while the years of education increase the number of days to reach the peak. As before, population density shows positive and significant coefficients for the peak of deaths regressions.

**Table 7:** Spatial regressions.

Variables	Days to peak (cases)	Peak of deaths				Days to peak (deaths)				
	(1)	(2)	(3)	(4)	(5)	(6)	(7)	(8)	(9)	(10)
Women		-9.456	23.377	-30.26	45.136	76.743	63.092	2.711	-25.066	
Children		-620.043**	-613.191**	-522.592*	-496.787	-285.822**	-259.304**	-100.258	-102.77	
People 65+		8.708	-23.345	-99.113	-153.403	-130.896***	-117.632**	-95.151*	-76.386	-45.941
People 80+		103.343	97.8213	165.106	134.547	65.410	72.103	142.891	158.972	
Migrants		-77.8217	-68.4965	-139.26	-107.79	-34.884	-35.264	-75.032*	-87.199**	
Population density	-0.0004	0.002**	0.002**	0.003***	0.002**	-4.43E-05	-7.62E-05	-4.45E-04	-3.17E-04	-0.0005*
Rurality		-70.224**	-73.757**	-49.2687	-60.4497	-74.930***	-74.644***	-68.746***	-65.216***	-79.102***
Multidimensional poverty	-8.085	101.431**	83.265*			-26.906	-24.657			-35.465*
Income poverty				-67.462	-94.926			67.6838	82.5887	
No overcrowding				44.953	52.752			-102.822**	-106.482**	
Critical overcrowding				464.011	431.904			-57.8263	-43.1727	
Years of education				4.0846	4.474			6.544**	6.460**	
Self-employed worker				228.15	177.625			-38.352	-21.033	
Green spaces		0.4810	0.384	0.482	0.329	0.0818	0.0564	-0.041	0.013	
Use public transportation	63.946**	-27.383	-68.326	-70.828	-141.338	-37.726	-28.510	-9.6124	16.709	
Water inside the house		-131.964	-104.831	7.413	59.075	48.668	31.673	27.682	1.949	
Public health insurance				68.686	62.570			10.194	12.184	
Difficulty getting healthcare	-24.329	1727.94	2189.74	1155.48	2209.2	342.868	282.777	-294.38	-667.431	
Distance to health center		-36.986	-36.69	-8.613	-8.224	-66.302***	-66.918***	-76.052***	-76.081***	-67.963***
COVID-19 risk factors		-131.877	-128.927	-85.627	-50.797	-29.793	-38.610	-29.862	-40.965	
Cumulative incidence			0.003		0.005		-0.0005		-0.0023	-1.69E-03
Spatial term	-0.342*	-0.459*	-0.477**	-0.601**	-0.667***	-0.745***	-0.625***	-0.539**	-0.469**	-0.546**
Constant	51.637***	249.626	213.952	-29.889	-115.976	117.311	138.781	179.811	217.897	74.778***
R-squared	0.166	0.516	0.523	0.547	0.570	0.693	0.674	0.712	0.706	0.614
Moran’s I (residuals)	-0.002	-0.142*	-0.143*	-0.111	-0.108	-0.099	-0.113	-0.203***	-0.183***	0.012

Significance level

*** p<0.01

** p<0.05

* p<0.1

## Discussion

Using municipality-level data on 52 administrative units, the article explored the effects of different sets of variables, acknowledging the need to consider a broad set of variables, as well as time dynamics and spatial effects in the analysis. Several conclusions are drawn from the results. First, there are common and idiosyncratic elements that explain the prevalence and dynamics of infections and mortality. It is necessary to recognize these different approaches when discussing the “impact” on COVID-19. The proposed models better explain variation in levels of infections and deaths than changes (measured as days to the peak) due to conceptual issues and measurement issues. Among conceptual issues, different variables affect different outcomes; research on the outcomes of COVID-19 needs to incorporate varied perspectives (measures of impact and determinants) to shed light on the problem. For measurement, due to data restrictions, the incidence variables are precise in capturing the underlying concept (scale of impact), while the use of incidence at the peak and days to the peak have a less clear interpretation. Better data could help improving these estimations by defining, for example, the share of cases within a given period, or the growth rate of cases.

Second, as long as different models are required to tell different stories (34 estimations in this case), a significant part of the municipal variation in infections and deaths can be explained by a rather small number of variables (as in Table 6). Results highlight the role of social determinants of health in explaining the dissimilar impacts of COVID-19 in the Metropolitan Region as well as other findings in other countries about unequal distribution of COVID-19 [37, 90–92]. In our study, the multidimensional poverty index informs COVID-19 infections and deaths, capturing the complex nature of the problem, highlighting the role played by structural determinants—particularly with poverty and vulnerability—and reflecting similar outcomes to other studies [93–96]. This result has more relevance considering that COVID-19 also has an impact on socioeconomic factors, generating a vicious circle between both problems [97–99]. Elucidating social determinants as an indicator of impact can inform policy response and future prevention efforts.

Third, the results identify different types of variables that explain the COVID-19 outcomes: demographic, health-related, and socioeconomic. However, these determinants can also be grouped according to their degree of changeability. Some of the relevant indicators can be seen—at least in the short run—as fixed (such as poverty and age distribution), while others are more flexible (like the use of public transportation and the availability of a health center). From a public policy perspective, this classification is useful: the first group of indicators can be assumed in the short-term as “explanatory” but be used for long-term planning while the second group of indicators are “policy tools” and can be used as control knobs to short-term responses to the pandemic.

### Limitations

The study’s limitations must be taken into account when interpreting the results. First, there are time-differences between dependent and independent variables: while dependent variables reflect information between March and July 2020, independent variables primarily originate from a survey carried out three years earlier. Unfortunately, there is no other municipality-level representative source of data with more updated information. However, most of the variables are expected to be similar today as aggregated data generally changes slower than individual-level data and most variables reflect structural factors—such as demographic and socioeconomic features—that are not expected to change significantly in a three-year period. “Policy tools” variables (such as the patterns of use of public transportation) experience similar change in absence of a policy shock.

Second, choosing an ideal methodology to measure the relevant outcomes can present a challenge as different specifications can lead to different results. Our analysis confronted this challenge by using several perspectives (infections and deaths, and levels and change). Moreover, although we use publically available, official reports [39–41] and secondary databases, previously utilized in other studies in Chile [5, 29], data could be still subject to some bias, as shown by the recent discussion around cases and death in the country [100]. In addition, we made methodological choices when selecting the unit of analysis focusing on municipalities and using percentages of individuals instead of, for example, households, to calculate the independent variables. Each choice has ramifications for interpretation. The small number of observations makes the precision of the inference difficult. We reported several models to show different perspectives and test different hypothesis. Also, the analysis does not delve into the scale of the reported coefficient; although several estimations allow comparisons between models, the meaning of the coefficients in each regression needs to be done carefully, particularly considering the presence of spillover effects in the spatial lag models, which depend on the degree of spatial correlation between the observations [86]. Consequently, interpretation of the results and their significance should follow a conservative criterion.

Finally, the definition of neighbors is crucial one since it could be driven the results; although in this case the selection of contiguous units is justified by the features of the data (the existence of units of different sizes and the spatial patterns of the variables of interest). The Metropolitan Region is a good case for spatial analysis but other geographical areas could also be of interest to understand the COVID-19 dynamics and policy responses (e.g. for establishing regional or international sanitary customs).

## Conclusions

This multi-perspective analysis of the COVID-19 impact in the Metropolitan Region of Chile highlights patterns and dynamics of the disease and the need to investigate social determinants of health and spatiotemporal dynamics in analyzing COVID-19 [90]. The study also prompts further research questions including comprehensive effects once the pandemic ends. We used multiple indicators—for example, a 100-day period to understand evolving factors—but recognize the analysis is a snapshot of an ongoing pandemic as well as our measure for the speed of change. We based our estimation on municipalities’ structural features; the understanding of COVID-19 outcomes can also improve by adding real-time variables related to people’s behavior, such as the percentage of people using facemasks, or better data on mobility within and between geographical areas. Overall, while improved data availability and quality can expound on changes in COVID-19 outcomes, we believe the analysis contributes to the worldwide effort for understanding the social determinants and effects of the COVID-19. Additionally, other spatial methods could be used to explore the data, as well as a deeper analysis of the scale of the effects presented.

We expect this study would be useful for policymakers in Chile, particularly in assessing future prevention and management strategies. In this line, the next generation of COVID-19 policies should, for example, take into account these results when designing quarantines and mobility restrictions (considering the spatial dynamics of the disease), defining people-at-risk and (considering the relevance of multidimensional poverty), and implementing short and long-term strategies (considering the role of public transportation). Additionally, we hope the results can encourage the implementation of evidence-based solutions and tailored interventions to help minimize the negative effects of the pandemic tackling social inequalities in other contexts.

## Supporting information

S1 Table Date used to establish the period of analysis in each municipality. (DOCX)

S2 Table Index of multidimensional poverty. (DOCX)
