# Supplementary material for: COVID-19 incidence and mortality in the Metropolitan Region, Chile: Time, space, and structural factors
Source: PLoS One. 2021 May 6;16(5):e0250707. doi: 10.1371/journal.pone.0250707 (PMC8101927; doi:10.1371/journal.pone.0250707)
Supplement: S1 Table — (DOCX) [file pone.0250707.s001.docx]

S1 Table. Date used to establish the period of analysis in each municipality

| Administrative unit (municipality) | Date of notification in national registry of first case by date of onset of symptoms | Day 100 | Number of days between the first case notified in the national registry and the day of the official report used for the analysis to day 100 |
| --- | --- | --- | --- |
| Alhué | 06-05-20 | 14-08-20 | 85 |
| Buin | 18-03-20 | 26-06-20 | 101 |
| Calera de Tango | 19-03-20 | 27-06-20 | 100 |
| Cerrillos | 21-03-20 | 29-06-20 | 101 |
| Cerro Navia | 17-03-20 | 25-06-20 | 102 |
| Colina | 10-03-20 | 18-06-20 | 100 |
| Conchalí | 17-03-20 | 25-06-20 | 102 |
| Curacaví | 19-03-20 | 27-06-20 | 100 |
| El Bosque | 19-03-20 | 27-06-20 | 100 |
| El Monte | 18-03-20 | 26-06-20 | 101 |
| Estación Central | 16-03-20 | 24-06-20 | 103 |
| Huechuraba | 14-03-20 | 22-06-20 | 100 |
| Independencia | 15-03-20 | 23-06-20 | 104 |
| Isla de Maipo | 27-03-20 | 05-07-20 | 104 |
| La Cisterna | 17-03-20 | 25-06-20 | 102 |
| La Florida | 10-03-20 | 18-06-20 | 100 |
| La Granja | 16-03-20 | 24-06-20 | 103 |
| La Pintana | 11-03-20 | 19-06-20 | 103 |
| La Reina | 11-03-20 | 19-06-20 | 103 |
| Lampa | 18-03-20 | 26-06-20 | 101 |
| Las Condes | 07-03-20 | 15-06-20 | 103 |
| Lo Barnechea | 08-03-20 | 16-06-20 | 102 |
| Lo Espejo | 16-03-20 | 24-06-20 | 103 |
| Lo Prado | 15-03-20 | 23-06-20 | 104 |
| Macul | 14-03-20 | 22-06-20 | 100 |
| Maipú | 16-03-20 | 24-06-20 | 103 |
| Maria Pinto | 14-04-20 | 23-07-20 | 100 |
| Melipilla | 14-03-20 | 22-06-20 | 100 |
| Ñuñoa | 05-03-20 | 13-06-20 | 101 |
| Padre Hurtado | 12-03-20 | 20-06-20 | 102 |
| Paine | 10-03-20 | 18-06-20 | 100 |
| Pedro Aguirre Cerda | 15-03-20 | 23-06-20 | 104 |
| Peñaflor | 13-03-20 | 21-06-20 | 101 |
| Peñalolén | 13-03-20 | 21-06-20 | 101 |
| Pirque | 11-03-20 | 19-06-20 | 103 |
| Providencia | 11-03-20 | 19-06-20 | 103 |
| Pudahuel | 16-03-20 | 24-06-20 | 103 |
| Puente Alto | 14-03-20 | 22-06-20 | 100 |
| Quilicura | 05-03-20 | 13-06-20 | 101 |
| Quinta Normal | 08-03-20 | 16-06-20 | 102 |
| Recoleta | 17-03-20 | 25-06-20 | 102 |
| Renca | 18-03-20 | 26-06-20 | 101 |
| San Bernardo | 17-03-20 | 25-06-20 | 102 |
| San Joaquín | 17-03-20 | 25-06-20 | 102 |
| San José de Maipo | 15-03-20 | 23-06-20 | 104 |
| San Miguel | 13-03-20 | 21-06-20 | 101 |
| San Pedro | 18-04-20 | 27-07-20 | 103 |
| San Ramón | 19-03-20 | 27-06-20 | 100 |
| Santiago | 28-02-20 | 07-06-20 | 100 |
| Talagante | 17-03-20 | 25-06-20 | 102 |
| Tiltil | 18-03-20 | 26-06-20 | 101 |
| Vitacura | 04-03-20 | 12-06-20 | 102 |
| Average |  |  | 101,69 |
