# Supplementary material for: COVID-19 incidence and mortality in the Metropolitan Region, Chile: Time, space, and structural factors
Source: PLoS One. 2021 May 6;16(5):e0250707. doi: 10.1371/journal.pone.0250707 (PMC8101927; doi:10.1371/journal.pone.0250707)
Supplement: S2 Table — (DOCX) [file pone.0250707.s002.docx]

S2 Table. Index of multidimensional poverty

| Dimension | Indicator | Weight | Rule of decision (household-level) |
| --- | --- | --- | --- |
| Education | School attendance | 7.5 | One member (ages 4 to 18) is not attending to school (except if already graduated), or at least one member aged 6 to 26 has a permanent and/or long-term condition and is not attending to school. |
|  | Scholarchip | 7.5 | At least one member (18+ years) old has less years of schooling than those established by law for her/ his age. |
|  | School lag | 7.5 | At least one member (21 or younger) is going to primary or secondary-level education and is two or more years lagged. |
| Health | Child Malnutrition | 7.5 | At least one of its members (0 to 6 years) has overweight or is obese, or is in malnutrition or at risk of malnutrition. |
|  | Health Insurance | 7.5 | At least one of its members has no health insurance. |
|  | Healthcare | 7.5 | At least one of its members did not receive healthcare in the last 3 months or was not covered by the AUGE-GES system (85 health problems with guarantees of access, opportunity, financial protection and quality), for reasons beyond his control or preference. |
| Labor and Social Security | Employment | 7.5 | At least one of its members (18+) is unemployed. |
|  | Social security | 7.5 | At least one of its members (15+) that is employed does not contribute to the pension system and is not a self-employed person with a complete higher education. |
|  | Pensions | 7.5 | At least one of its members at retirement age does not receive a contributory or non-contributory pension and does not receive other income from rentals, profit withdrawals, dividends and interest. |
| Housing and environment | Housing conditions | 7.5 | (a) Are in a situation of overcrowding (the number of persons in the household per exclusive use bedroom is greater than or equal to 2.5); or, (b) Lives in a precarious dwelling or in a dwelling with walls, ceilings and/or floor in poor condition. |
|  | Basic services | 7.5 | Living in a house without basic sanitary services (WC, key inside the house and water according to urban or rural standards). |
|  | Environment | 7.5 | (a) Identifies 2 or more environmental pollution problems that frequently occur in the area of residence; or, (b) have no occupied members and lack in their area of residence any of the three basic facilities (health, education, and transportation); or, (c) lack in their area of residence any of the three basic facilities (health, education, and transportation) and have occupied members who use public or non-motorized transportation and on average take 1 hour or more to get from their home to their main place of work. |
| Networking and social cohesion | Social support and participation | 3.33 | Do not have any person who can help (outside of the household members) in 8 relevant support or care situations; nor do they have members 14 years old or older who have participated in the last 12 months in any social organization or group and nor do they have members 18 years old or older who are employed and belong to any organization related to their work / total number of households. |
|  | Equal treatment | 3.33 | Declares that any of its members has been discriminated against or treated unfairly during the past 12 months for any of the reasons listed in the respective question |
|  | Security | 3.33 | Households that state that one of their members has "always" lived or witnessed, during the last month, at least one of the following situations in their area of residence: i. Drug trafficking; or, ii. Shooting |

Source: Ministry of Social Development, Casen Survey 2015-2017.
